# Lessons for Humanities and Arts in Gerontology and Geriatrics Curricula From the Medical and Health Humanities

**DOI:** 10.1093/geroni/igab046.267

**Published:** 2021-12-17

**Authors:** Desmond O'Neill

**Affiliations:** Trinity College Dublin, Dublin, Dublin, Ireland

## Abstract

The role of Humanities, Arts and Cultural Gerontology in gerontology and geriatrics curricula finds a metaphor in the rapidly evolving field of medical and health humanities, with which this author has been involved for three decades. Behind the call for increasing humanities and arts scholarship in the pedagogy of both fields lies the challenge of establishing an interdisciplinary nexus of scholarship that avoids the challenges of dilettantism and gestures such as providing lists of novels and movies. This presentation draws on the presenter's bibliometric research in the medical and health humanities which indicates authorship in the majority to be either solely from the humanities or from healthcare, with little indication of joint working in either authorship or acknowledgements (the scholar's courtesy), and explores the background issues of academic culture with a view to proposing solutions to elevate the inclusion of humanities and arts as a significant element of gerontology education.

